# Allergic contact dermatitis of both eyes caused by alcaftadine 0.25%: a case report

**DOI:** 10.1186/s12886-019-1166-2

**Published:** 2019-07-24

**Authors:** Jae Hyuk Kim, Hyun Joon Kim, Sun Woong Kim

**Affiliations:** Department of Ophthalmology, Wonju Severance Christian Hospital, Wonju, South Korea

**Keywords:** Alcaftadine 0.25%, Allergic contact dermatitis, Allergic conjunctivitis

## Abstract

**Background:**

To report the first case of allergic contact dermatitis (ACD) associated with alcaftadine 0.25% ophthalmic solution.

**Case presentation:**

The patient was a 51-year-old woman with no previous history of side effects to ophthalmic antihistamine agents. She had been prescribed alcaftadine 0.25% for allergic conjunctivitis. On first application of the medication, she did not experience any cutaneous reaction. One day later, after the second alcaftadine 0.25% application, both eyelids became swollen, and erythematous changes were evident. On slit-lamp examination, conjunctival injection was noted in the absence of conjunctival swelling or any other findings. Fundus examination was unremarkable. To evaluate the cause of ACD, a patch test was performed and 48 h later was noted to be positive for alcaftadine 0.25%. Based on the positive patch test, the patient was diagnosed with ACD caused by alcaftadine 0.25%. After 9 days of treatment, the swelling and erythema completely resolved.

**Conclusions:**

Although there have been no previous reports of alcaftadine 0.25%-associated ACD, it should be suspected in patients with swelling and erythematous change of both eyes after using alcaftadine 0.25%.

## Background

Contact dermatitis is one of the most common skin diseases and is an inflammatory skin condition induced by exposure to environmental agents [1]. Skin is the first barrier against chemical and physical factors in the environment. There are two types of contact dermatitis: irritant contact dermatitis, and allergic contact dermatitis (ACD). Irritant contact dermatitis is due to toxic effects of chemical or physical factors that activate the skin’s innate immunity. However, ACD requires the activation of antigen-specific acquired immunity leading to the development of effector T cells, which mediate the skin inflammation [2].

The most common causes of ACD are minerals such as nickel, chromium, cobalt, gold and organic chemicals [3]. In Korea, lacquer [4], rubber [5], hair dye [6], minerals (such as nickel, chromium, cobalt, and mercury), and cosmetics [7] are the main causes of ACD. Those chemicals work as haptens, which induce an immune response only when attached to larger molecules. The haptens can pass through the skin and reach the local lymph node, and effector T cells are then formed. The pathophysiology of ACD consists of two distinct phases. Phase 1 is called the induction phase. This occurs at the first contact between skin and haptens and leads to the generation of effector T cells. After phase 1, phase 2, the elicitation phase, is induced in sensitized individuals when challenged by the same haptens. Haptens diffuse in the skin and are taken up by skin cells, which leads to the activation of effector T cells in the dermis and epidermis. This triggers the inflammatory process responsible for the cutaneous lesions and occurrence of ACD [2, 8].

Currently, ACD is diagnosed by performing a patch test. The patch test is used to detect the causative contact allergens and indicates contact sensitization of past or present relevance. The patch test does not produce a false-positive reaction, and is now the universally accepted method for ACD [2].

Antihistamine medications are frequently prescribed to treat allergic reactions [9]. In the United States, alcaftadine 0.25% ophthalmic solution (Lastacaft®; Allergan, Inc., Irvine, CA, USA) has been approved for the prevention of itching associated with allergic conjunctivitis [10]. Its effectiveness for allergic conjunctivitis and safety are well studied [11]. Previous reports indicated that fewer than 4% of patients experience adverse effects such as ocular irritation, pruritus, erythema, and stinging or burning upon instillation [12, 13]. However, there are no reports of alcaftadine 0.25% causing ACD. This is the first report of ACD diagnosed after the use of alcaftadine 0.25% resulted in swelling around both eyes with erythematous changes.

## Case presentation

**Fig. 1:**
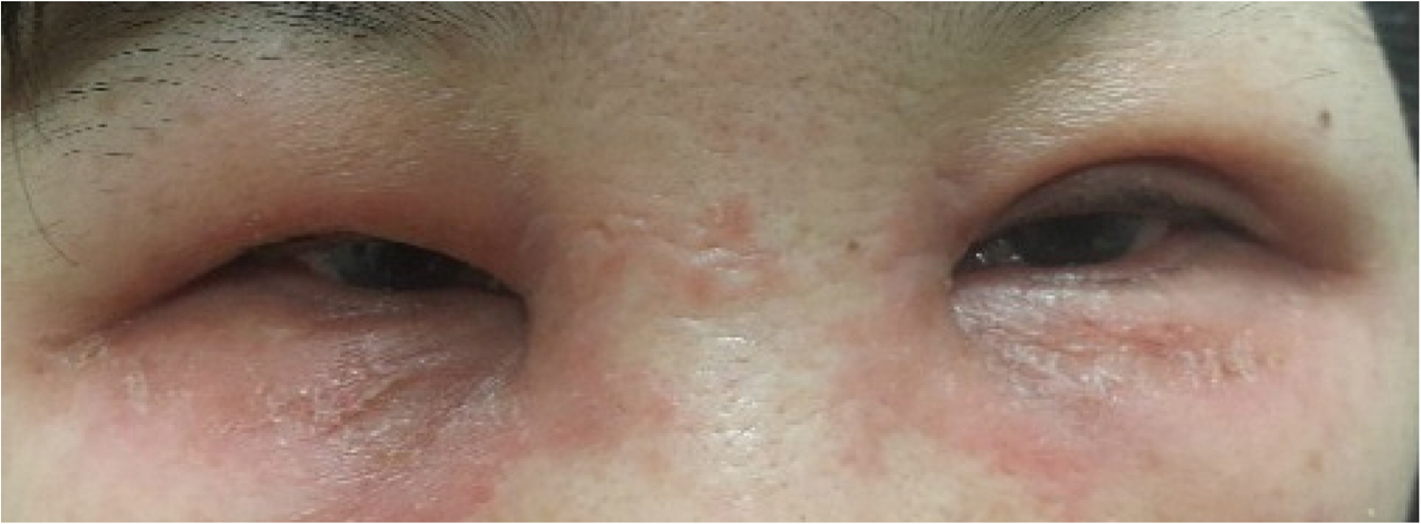
Physical examination at initial presentation. Severe eyelid swelling of both eyes with erythematous change

**Fig. 2:**
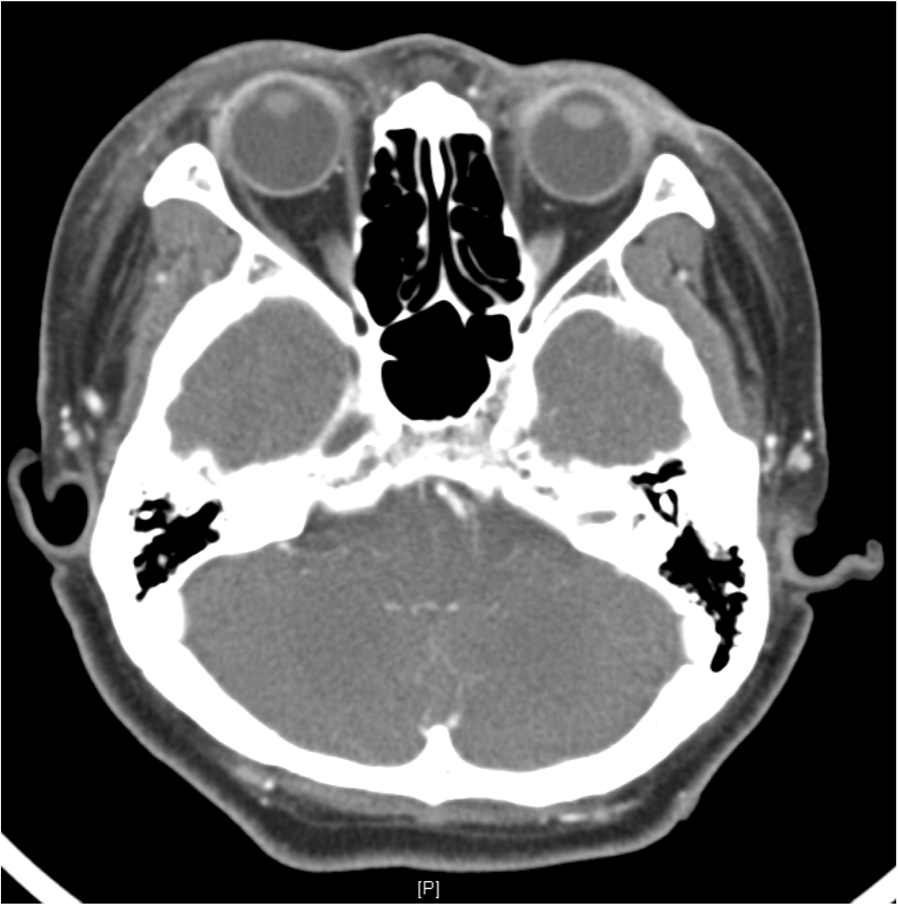
Facial computed tomography image showing no signs of inflammation near the orbits

The patient was a 51-year-old woman with no previous history of allergy. She presented to the emergency room for bilateral severe eyelid swelling for 1 week. On eye examination, visual acuity was 20/25 for both eyes and intraocular pressure was 14 mmHg bilaterally. Slit-lamp examination was unremarkable except for conjunctival injection without conjunctival swelling. Fundus examination was also unremarkable. Severe eyelid swelling of both eyes was noted (Fig. 1). To rule out orbital cellulitis, facial computed tomography (CT) was performed in emergency department. On facial CT, no signs of inflammation near orbits were found (Fig. 2).

Two weeks previously, the patient had visited the local clinic with symptoms of eye congestion, gritty feeling, and tearing. She was diagnosed with conjunctivitis and was prescribed levofloxacin 0.5% (Cravit®; Santen Pharm. CO., Japan) and fluorometholone 0.1% (Fumelon®; Hanlim Pharm. CO., LTD., South Korea), with no improvement. One week later, she was prescribed alcaftadine 0.25% by a local physician based on a presumptive diagnosis of allergic conjunctivitis. The patient said eyelid swelling with erythematous changes in both eyes started the day after starting alcaftadine 0.25%.

**Fig. 3:**
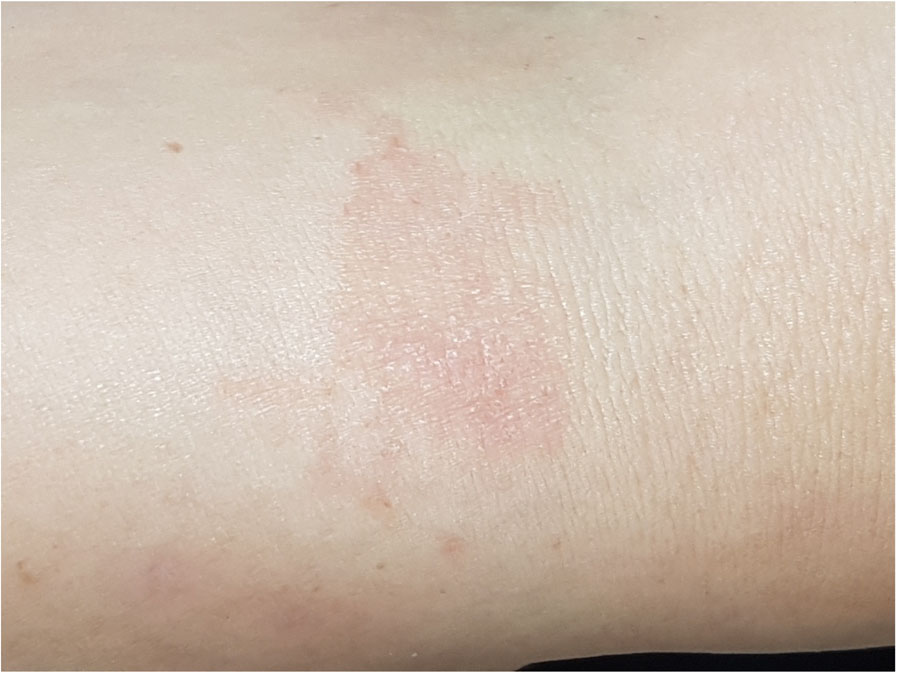
Patch test results. Erythematous change was found only on site of alcaftadine 0.25%

**Fig. 4:**
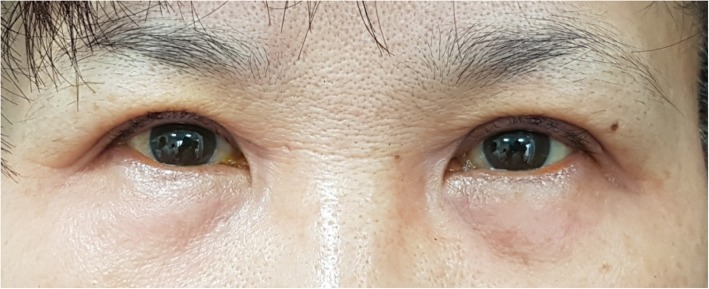
Physical examination after treatment. Eyelid swelling has resolved

When she was referred to our department, we suspected ACD and consulted with dermatology for evaluation of ACD; the patient was instructed not to use alcaftadine 0.25%. To evaluate ACD and to find the cause, a patch test was performed on her forearm. The three ophthalmic agents the patient had used were applied and covered with Tegaderm™. She was prescribed only oral steroids for treatment of the swelling and erythema. There was no topical steroid used. After 2 days, patch test results showed well-bordered erythematous lesion which was only on the area where alcaftadine 0.25% had been applied (Fig. 3). It was confirmed by our hospital’s dermatologist. Therefore, the diagnosis of alcaftadine 0.25%-associated ACD was confirmed. On eye examination, there was no specific change and no specific ophthalmic problems, although the severe eyelid swelling remained. Oral steroids were maintained **as 8 mg of methylprednisolone**. One week after discontinuing alcaftadine 0.25%, the eyelid swelling was remarkably improved. She was instructed to continue oral steroids for two more weeks with dose tapering **as 4 mg of methylprednisolone**. Nine days after discontinuation of alcaftadine 0.25%, the eyelid swelling had vanished, and conjunctival injection had disappeared (Fig. 4). The patient was prescribed artificial tears and followed up for **2** months, and there was no other event thereafter.

## Discussion and conclusions

The patient had no previous history of side effects related to ophthalmic antihistamine agents. When the patient first applied alcaftadine 0.25%, there was no cutaneous reaction. One day later, after the second application of alcaftadine 0.25%, bilateral eyelid swelling with erythematous changes was noted. To evaluate the cause of ACD, a patch test was performed. The result was checked 48 h later; Among 3 ophthalmic agents applied, only alcaftadine 0.25% showed a positive result. Although we did not test all components individually, other ingredients such as benzalkonium chloride, monobasic sodium phosphate, dibasic sodium phosphate, sodium chloride, and sodium hydroxide are also included in other two eyedrops we tested. We could exclude a possibility of irritation because skin lesion remained for more than 1 week, while skin lesions induced by irritant typically disappear within 96 h after patch is removed (ref). Therefore, based on the results of the patch test, the patient was diagnosed with ACD caused by alcaftadine 0.25%.

Drug allergy is a type B reaction that is mediated by the adaptive immune system. Type B reactions are uncommon and unpredictable and occur only in people with a certain predisposition. However, recent reports show that previous contact with the causative drug is not a prerequisite for immune-mediated drug hypersensitivity [14]. Sensitization is possible either when are drugs applied to the skin or administered orally. Patients may become more sensitized to antihistamine agents when they are applied to the skin rather than taken orally [15]. In the present case, the patient used alcaftadine 0.25%, which is an antihistamine agents for the treatment of allergic conjunctivitis. Presumably, the eyelid skin was exposed when the drug overflowed and erythematous changes developed by allergic response.

Currently, alcaftadine 0.25% is one of the most prescribed antihistamine agents for allergic conjunctivitis, and is used worldwide [10, 11, 16, 17]. Alcaftadine 0.25% is an H_1_- and H_2_-receptor antagonist [11]. There are some reports of side effects related to alcaftadine 0.25% usage [10, 11, 17], and previous studies reported fewer than 4% of subjects had side effects [12, 13]. Its side effects are usually related to ocular problems such as ocular itching, conjunctival redness, chemosis, lid swelling, and tearing [10, 11, 17]. An atypical symptom of bronchitis was also reported [17]. However, there have been no reports to date that alcaftadine 0.25% induces ACD.

There have been some reports of ACD induced by antihistamine agents [18]. Histamine can have direct effects on T lymphocytes, as H_1_, H_2_, and H_4_ receptors are all expressed on CD4^+^ and CD8^+^ T cells. Histamine has been shown to inhibit T-cell proliferation through H_2_ receptors [15]. Therefore, ACD induced by antihistamine agents can be explained by T-cell proliferation caused by inhibiting histamine to H_2_ receptors [19–21]. However, not all antihistamine agents can induce ACD. This is because the effect of histamine on T-cell proliferation seems to vary. Histamine also can increase T-cell proliferation through H_1_ receptors. In this case, antihistamine agents reduce allergic symptoms by inhibiting T-cell proliferation mediated through H_1_ receptors. Therefore, histamine has a key role in inflammatory processes, and drugs that target H_1_ receptors have usually been successful for the treatment of allergy [15]. However, it is important to note that when using antihistamine agents with strong affinity to H_1_ and H_2_ receptors, such as alcaftadine 0.25%, ACD may occur [11].

Ophthalmic agents can cause various side effects and there are many explanations including drug-related allergies, toxic or inflammatory reaction of the drugs, and toxicity of preservatives such as benzalkonium chloride [17, 22]. However, the concentration of benzalkonium chloride in alcaftadine 0.25% is 0.005%, which is very low, and other ophthalmic agents also have approximately the same concentration [22]. Considering that the other ophthalmic agents she used, which also contained benzalkonium chloride, did not induce any allergic reaction, we could attribute alcaftadine 0.25% itself as the causative factor of allergic reaction.

In the present case report, we report a patient with ACD after using alcaftadine 0.25%, which was so severe that the patient required treatment for 9 days until it resolved completely. There is a possibility that more cases of ACD have occurred after using alcaftadine 0.25%, even though no previous reports have been published.

From now on, ophthalmologists should inform patients of the possibility of ACD when prescribing alcaftadine 0.25%. We suggest that wiping off run-over may be useful to prevent this side effect. This case report suggests when cutaneous reactions near the orbit are found after alcaftadine 0.25% use, ophthalmologists should consider ACD.

## Data Availability

Not applicable

## Abbreviations

ACD: Allergic contact dermatitis
CT: Computed tomography
